# 256 Bridging the Gap: Translating Sport Injury Prevention Research into Community-Level Practice

**DOI:** 10.1093/eurpub/ckae114.280

**Published:** 2024-09-26

**Authors:** Theresa Heering, Natalie Lander, Jason Tallis, Lisa Barnett, Michael Duncan

**Affiliations:** Coventry University, UK; Deakin University, Australia; Deakin University, Australia; Coventry University, UK; Deakin University, Australia; Coventry University, UK

**Keywords:** risk reduction, children, sport injury

## Abstract

An anterior cruciate ligament (ACL) injury is a severe musculoskeletal injury with significant consequences for individuals (e.g., long rehabilitation periods and an increased risk of reinjury), as well as for the healthcare system (e.g., high treatment costs). In developed countries such as Australia and Norway, the prevalence of ACL injuries has been increasing among children aged 5-14. Therefore, appropriate and effective risk reduction strategies are urgently needed. However, effective risk reduction is often hindered by a lack of knowledge or involvement from stakeholders, particularly in youth sports at the community level.

Addressing this gap, the current project aims to bridge the gap between sport injury prevention research and community-level practice. It seeks to translate findings from my recent PhD research into actionable strategies for community sports stakeholders, including sport clubs, coaches, parents, and athletes.

Using “The Physical Activity Messaging Framework” by Williamson et al. (2021), a communication strategy was developed, resulting in two tailored reports. The first report, designed for pediatric grassroots footballers, presents information on injury prevention in an accessible, “fun” format suitable for children. The second report, aimed at the parents and coaches of these young athletes, provides more in-depth information on sport injury prevention, such as focusing on improving landing techniques. Both reports were piloted with the target population (pediatric grassroots footballers: n = 4, and parents: n = 4) and adjusted according to their feedback. The final reports will be distributed to the target population (youth grassroots football players (n = 35), their parents (n = 70), and coaches (n = 5)) in September 2024.

The potential impact of this project includes promoting sport injury prevention practices through education, fostering conversations with coaches, parents, and athletes to increase awareness of ACL injuries in youth sports, and providing practical advice on reducing the risk of sporting injuries. This project not only addresses a crucial gap in sport injury prevention but exemplifies the practical application of research, ultimately contributing to safer sports environments at the community level.

